# Evidence of Different Thermoregulatory Mechanisms between Two Sympatric *Scarabaeus* Species Using Infrared Thermography and Micro-Computer Tomography

**DOI:** 10.1371/journal.pone.0033914

**Published:** 2012-03-19

**Authors:** José R. Verdú, Javier Alba-Tercedor, Mónica Jiménez-Manrique

**Affiliations:** 1 I.U.I. Centro Iberoamericano de la Biodiversidad (CIBIO), Universidad de Alicante, San Vicente del Raspeig, Alicante, Spain; 2 Departamento de Zoología, Facultad de Ciencias, Universidad de Granada, Campus de Fuentenueva, Granada, Spain; 3 Facultad de Ciencias, Universidad de Alicante, San Vicente del Raspeig, Alicante, Spain; University of Sydney, Australia

## Abstract

In endotherms insects, the thermoregulatory mechanisms modulate heat transfer from the thorax to the abdomen to avoid overheating or cooling in order to obtain a prolonged flight performance. *Scarabaeus sacer* and *S. cicatricosus*, two sympatric species with the same habitat and food preferences, showed daily temporal segregation with *S. cicatricosus* being more active during warmer hours of the day in opposition to *S. sacer* who avoid it. In the case of *S. sacer*, their endothermy pattern suggested an adaptive capacity for thorax heat retention. In *S. cicatricosus*, an active ‘heat exchanger’ mechanism was suggested. However, no empirical evidence had been documented until now. Thermographic sequences recorded during flight performance showed evidence of the existence of both thermoregulatory mechanisms. In *S. sacer*, infrared sequences showed a possible heat insulator (passive thermal window), which prevents heat transfer from meso- and metathorax to the abdomen during flight. In *S. cicatricosus*, infrared sequences revealed clear and effective heat flow between the thorax and abdomen (abdominal heat transfer) that should be considered the main mechanism of thermoregulation. This was related to a subsequent increase in abdominal pumping (as a cooling mechanism) during flight. Computer microtomography scanning, anatomical dissections and internal air volume measurements showed two possible heat retention mechanisms for *S. sacer*; the abdominal air sacs and the development of the internal abdominal sternites that could explain the thermoregulation between thorax and abdomen. Our results suggest that interspecific interactions between sympatric species are regulated by very different mechanisms. These mechanisms create unique thermal niches for the different species, thereby preventing competition and modulating spatio-temporal distribution and the composition of dung beetle assemblages.

## Introduction

Thermoregulatory mechanisms affect not only the internal functioning of an insect but also its activity (daily activity, phenology, diapause, overwintering, etc.), which is an important determinant of its relationship with the environment [1]. Measurements of body temperatures in the field, or under laboratory conditions closely mimicking field conditions, are often necessary to give an accurate picture of normal thermal behaviour and the extent of thermoregulation in insects [2]. When extreme ambient temperatures affect the physiological performance of an individual (or population, or species, etc.), the term ‘capacity adaptation’ is applied to explain the existence of different thermoregulatory mechanisms [3], [4].

In heterotherm insects, thermoregulatory mechanisms extend flight activity at both maximum and minimum suboptimal thoracic flight thermal temperatures (T_thorax min_ of take-off and T_thorax max_ to landing-overheating), which enhances flight performance. This behaviour has evolutionary implications and has been used to explain several cases of interspecific competition and/or thermal niche segregation [5], [6]. Flying insects need to have high metabolic rates, thereafter, so endogenous heat generation during flight implies the existence of heat loss and heat gain (or maintenance) mechanisms that prolong the equilibrium of T_thorax_ with ambient temperature (T_ambient_) during flight [7]. Heat loss during flight occurs via convective cooling (external mechanism), via the control of hemolymph circulation (internal mechanism) between the thorax and abdomen (by abdominal pumping) and, in some cases, by via respiratory evaporation (internal mechanism) [7]. On the contrary, heat gain or maintenance implies the existence of anatomical adaptions that avoid the heat loss such as thoracic piles, and a well-developed complex of abdominal and thoracic air sacs, which retard the passive conduction of heat from the thorax to the abdomen [7], [8].

**Figure 1:**
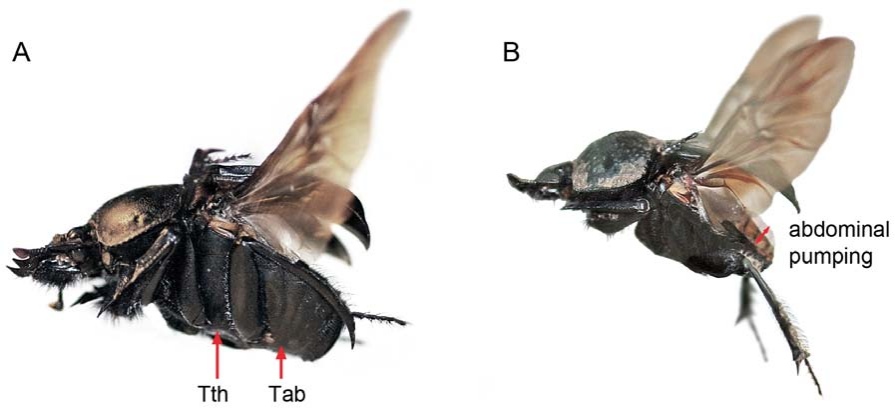
Flight postures of *Scarabaeus* species. (A) *S. sacer* and (B) *S. cicatricosus*. Arrows indicate regions in which body temperatures were analysed (T_thorax_ and T_abdomen_) and the abdominal movement to generate heat flow (abdominal pumping). The flight posture adopted by *S. cicatricosus*, with the posterior legs extended from the body, increase turbulence and convective cooling.

While interspecific and intraspecific competition occurs only occasionally for most dung beetles, in the case of rollers (dung beetles that remove a portion of dung from the mass, roll it some distance away from the source and then bury it), pairwise competitive interactions of individuals seem to be relatively frequent (for a review, see [9]). It has been demonstrated that thermoregulation is directly related to dung beetle behaviour in accordance with particular ecological requirements, being an essential element to explain the relevance of competition and/or segregation dynamics among species [6],[8],[10],[11],[12],[13]. In a previous study the two unique closely related sympatric roller dung beetle species inhabiting the Doñana National Park (Huelva, S-Spain) show a daily segregation of flight activity as consequence of different thermoregulatory strategies [11]. In *Scarabaeus sacer* Linnaeus, the thorax is the main heat generation centre and the thermoregulation is limited by the use of the abdomen as a ‘passive thermal window’; abdomen does not actively dissipate the increase of T_thorax_ generated during flight, thus increasing the danger of overheating when T_ambient_ oscillates around 35–40°C. On the contrary, *Scarabaeus cicatricosus* (Lucas) would actively thermoregulate its body temperature by transferring the excess of thoracic heat to the abdomen in order to dissipate it from there. This mechanism facilitates flying during the hottest period of the day avoiding reaching heat shock temperatures which are established at approximately 42°C for endothermic dung beetles [6]. The proposed mechanisms for this cooling strategy in *S. cicatricosus*, are related with the characteristics of abdominal intertergal cuticle sections and/or the adopted flight posture which increase convective cooling (see Figure 1; [11]); however, there are few evidences supporting these mechanisms [6].

This study provides empirical data showing that both thoracic and abdominal dung beetle body temperatures (T_thorax_ and T_abdomen)_ are sensitive to the mechanisms of heat transfer from the thorax to the abdomen during flight. This could further allow us to assess the existence of different thermoregulatory mechanisms [11]: abdominal passive heat transfer (APHT) and abdominal active heat transfer (AAHT). In the case of APHT we hypothesize that: i) T_thorax_ should increases independently of T_abdomen_; ii) the slope of T_abdomen_ against time should be similar to those T_ambient_ (if T_ambient_ is constant, T_abdomen_ also should be also approximately constant); iii) the difference T_thorax_–T_abdomen_ reflecting body temperature gradient should increase during flight time and its magnitude should be greater than those detected in the case of AAHT species. Contrariwise, in a species with an AAHT thermoregulatory mechanism we anticipate that i) variation in T_thorax_ will be correlated with T_abdomen_; ii) the slope of T_abdomen_ against time should be significantly different to those T_ambient_ (if T_ambient_ is constant, T_abdomen_ should have a different regression slope); iii) the difference T_thorax_ – T_abdomen_ should remain relatively constant along time. To test these hypotheses, we used infrared thermography, a technique that circumvents the problems associated with thermocouples measurements: the invasive character of the measurements (each individual datum comes from an injured beetle), the time needed to record each T_thorax_ and T_abdomen_ value for an individual, and the risk of overestimating T_thorax_ [14]. With high-resolution infrared thermography, the continuous recording of thermal behaviour of an undamaged beetle is possible and detailed thermal images of all body regions can be easily analysed.

## Results and Discussion

**Figure 2:**
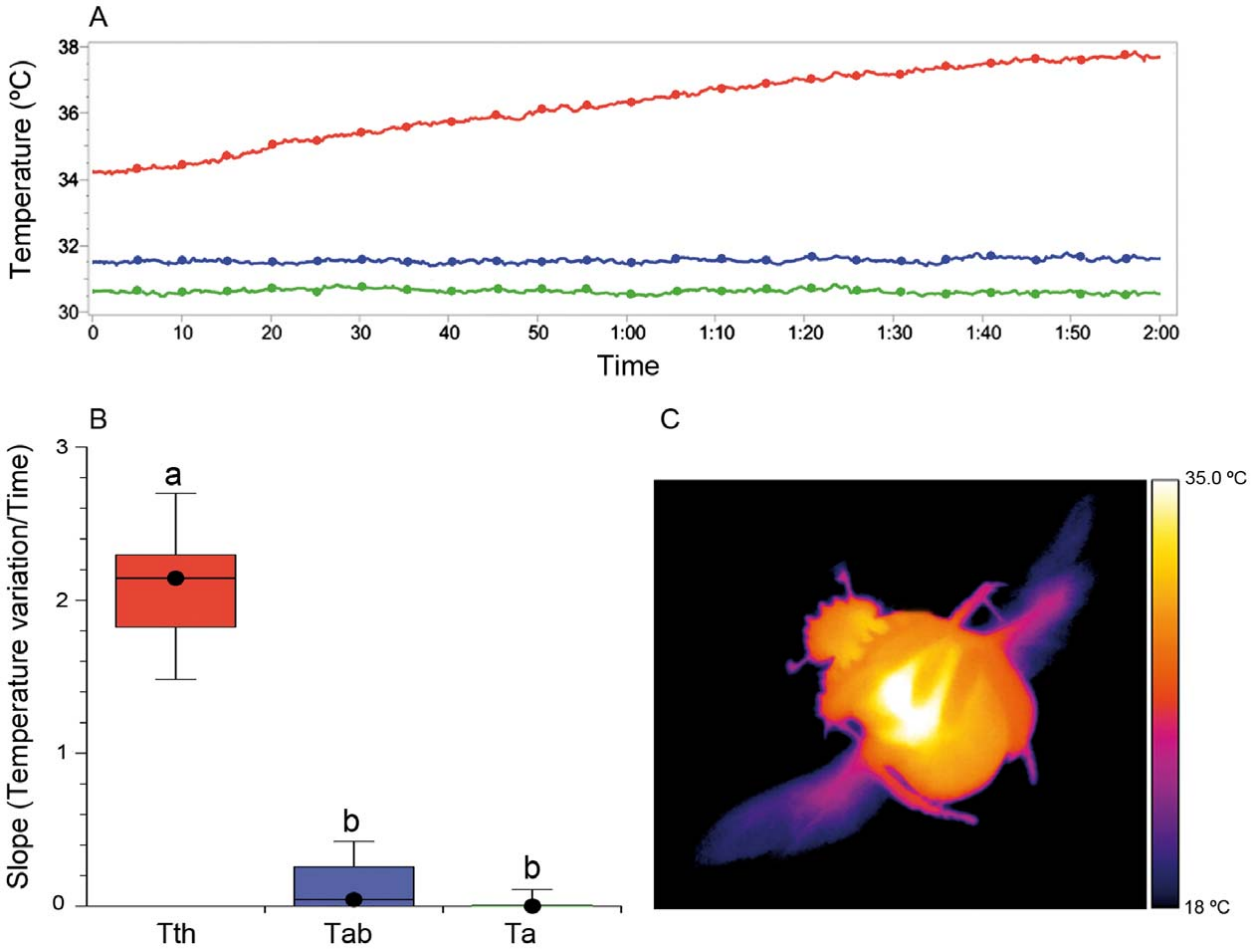
Flight thermoregulatory behaviour of *Scarabaeus sacer*. (A) Individual variation of T_thorax_ (in red) and T_abdomen_ (in blue). Environmental temperature (T_ambient_) (in green) was maintained during each experiment. (B) Comparison of slopes of T_thorax_, T_abdomen_ and T_ambient_ variation during flight (*N* = 27; Kruskal-Wallis test with the Conover-Inman post hoc test for pairwise comparisons, α<0.05); black dots indicate the median values; temperatures with the same letter did not differ significantly from each other. (C) Thermal image of *S. sacer* during flight showing the strong contrast between T_thorax_ and T_abdomen_.

**Figure 3:**
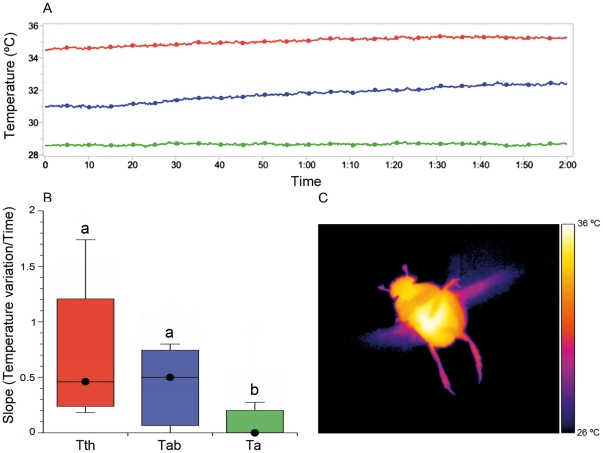
Flight thermoregulatory behaviour of *Scarabaeus cicatricosus*. (A) Individual variation of T_thorax_ (in red) and T_abdomen_ (in blue). Environmental temperature (T_ambient_) (in green) was maintained during each experiment. (B) Comparison of the slopes of T_thorax_, T_abdomen_ and T_ambient_ variation during flight (*N* = 27; Kruskal-Wallis test with the Conover-Inman post hoc test for pairwise comparisons, α<0.05); black dots indicate the median values; temperatures with the same letter did not differ significantly from each other. (C) Thermal image of *S. cicatricosus* during flight, showing lower contrast between T_thorax_ and T_abdomen_, relative to *S. sacer*.

Thermographical techniques demonstrated the existence of the two thermoregulatory mechanisms suggested by Verdú et al. [11]: a passive thermal window for *Scarabaeus sacer* and an active thermoregulatory abdominal mechanism for *S. cicatricosus*. Flight median temperatures (median and range) measured in the laboratory for the studied species were as follow: For *S. sacer*: T_thorax_ = 32.9°C (30.2 to 34.2°C), T_abdomen_ = 29.9°C (28.3 to 30.8°C) and T_ambient_ = 26.9°C (21.3 to 28.5°C). For *S. cicatricosus*, T_thorax_ = 34.9°C (32.3 to 36.6°C), T_abdomen_ = 32.0°C (29.9 to 33.2°C) and T_ambient_ = 29.9°C (25.7 to 31.3°C). According to our expectations, the slopes of thoracic and abdominal temperatures against time of *S. sacer* were significantly different (Kruskal-Wallis test *T* = 16.980; df = 2; *P*<0.001; Conover-Inman post-hoc test between T_thorax_ and T_abdomen_: *t* = 2.080; *N* = 24; *P*<0.0001). However, they were not statistically different in the case of *S. cicatricosus* (Kruskal-Wallis test *T* = 11.919; df = 2; *P*<0.001; Conover-Inman post-hoc test between T_thorax_ and T_abdomen_: *t* = 2.063; *N* = 27; *P* = 0.272). The individual linear slopes of T_abdomen_ against time were not significantly different from T_ambient_ slope in the case of *S. sacer* (Supporting information S1). However, for *S. cicatricosus* the individual linear slopes of T_abdomen_ against time were significantly different from those T_ambient_ (Supporting information S1). For *S. sacer*, the occurrence of significant differences between the slopes of T_thorax_ and T_abdomen_ during flight, and subsequently, the similitude between T_abdomen_ and T_ambient_ slopes confirm our previous hypotheses (see introduction section and Figure 2A and B). These data, in combination with the small difference between T_abdomen_ and T_ambient_, corroborate the evidence for the existence of a mechanism that prevents heat flow from the thorax to the abdomen. Thermographical images suggested the existence of a possible heat insulator, which hindering the heat transfer from meso- and metathorax to the abdomen (Figure 2C). This mechanism, called “passive thermal window”, has been observed in *S. sacer* during flight at a range of T_ambient_ oscillating from 18.2 to 34.5°C. However, this mechanism could be less effective at T_ambient_ above 30°C when abdominal temperature excess tend to decrease [11]. This behaviour permits *S. sacer* to fly during the coolest periods of the day, but may be hazardous at higher T_ambient_ values due to the possibility of the thorax overheating during flight [11]. Thus, this behaviour may require both a high endothermic capacity and the existence of mechanisms capable of preventing cooling during flight. The first condition was reported by Verdú et al. [11]. They showed that the increments of T_thorax_ in relation to T_ambient_ (T_excess_) were around 16°C in *S. sacer*. On the contrary, very small differences between T_thorax_ and T_abdomen_ during flight were observed in *S. cicatricosus* being also similar the slopes of T_thorax_ and T_abdomen_ against T_ambient_ (Figure 3A and B). All these evidences highly suggest the existence of heat flow between thorax and abdomen. Under this scenario, a higher heat flow between the thorax and the abdomen could explain the low difference between T_thorax_ and T_abdomen_ observed in thermal images (Figure 3C).

**Figure 4:**
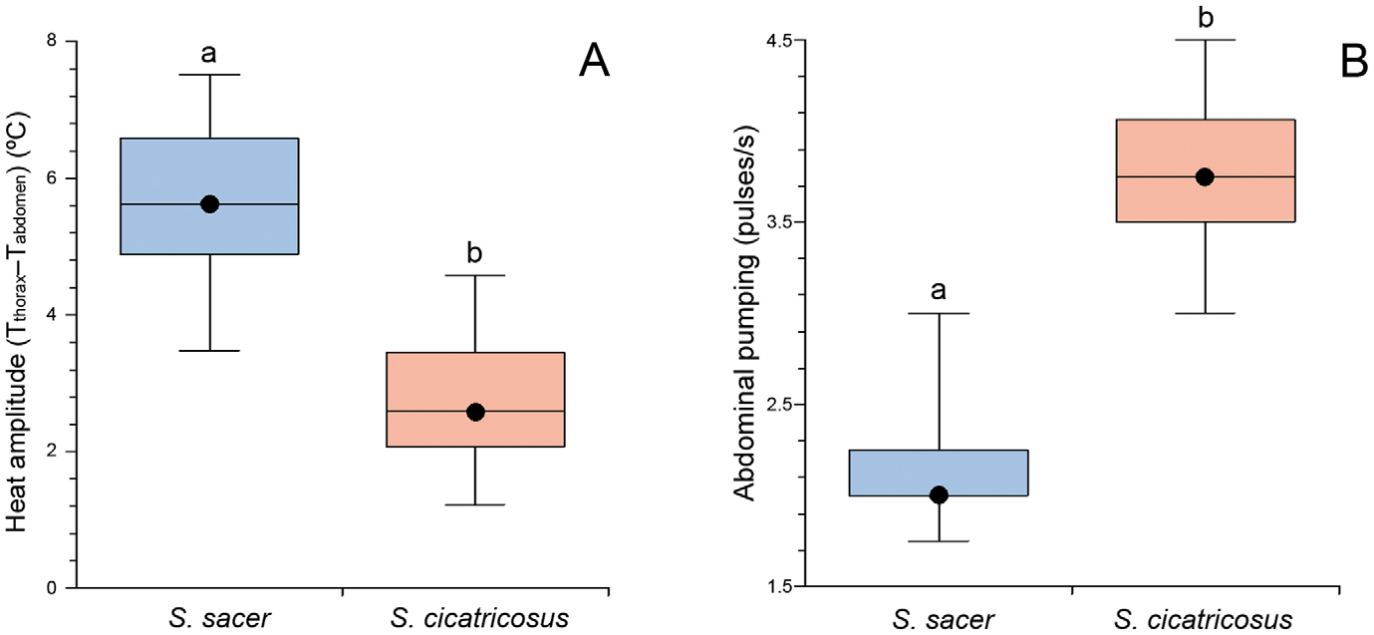
Heat transfer between the thorax and the abdomen and abdominal pumping behaviour. Differences between (A) T_thorax_ and T_abdomen_ during flight for *Scarabaeus sacer* (in blue) and *S. cicatricosus* (in red), and (B) frequency of abdominal pumping. (*Scarabaeus cicatricosus*, N = 27; and *S. sacer*, N = 24; U-Mann-Whitney, α<0.05); Boxes labelled with the different letters differ significantly from each other. Black dots indicate the median values.

In support of hypothesis iii, significant differences between T_thorax_ and T_abdomen_ during flight were observed in *S. sacer* and *S. cicatricosus* (*U* = 1415, *P*<0.0001; Figure 4A). *Scarabaeus sacer* showed a difference between T_thorax_ and T_abdomen_ of 5.6°C in average, whereas *S. cicatricosus* showed differences between T_thorax_ and T_abdomen_ of 2.6°C in average. Regarding cooling mechanisms, we have observed a low frequency of abdominal pumping, which is related to the higher amplitude of the temperature difference between the thorax and the abdomen compared to *S. cicatricosus* (Figure 3A and Figure 4A). In general, abdominal pumping, as a cooling mechanism, was significantly different between both species (*U* = 0.5, *P*<0.0001; Figure 4B). *Scarabaeus sacer* showed a slower frequency of abdominal pulses (2.0 pulses/s) compared to *S. cicatricosus* (3.8 pulses/s). For *S. cicatricosus*, thermoregulation during flight is very different; an effective mechanism of heat transfer between the thorax and the abdomen that permits flight performance during prolonged time at high T_ambient_. According to previous data [11], this ‘heat exchange’ mechanism observed in *S. cicatricosus* could be facilitated by the irregular posture adopted during flight, with the posterior legs extended from the body (see Figure 1B), which increases turbulence and convective cooling. In our results, however, thermography reveals clear and effective heat flow between the thorax and the abdomen and this should be considered the main mechanism of thermoregulation related to a subsequent increase in abdominal pumping during flight. This hypothesis was supported by the increase in abdominal temperature, which more effectively explains the increase in pumping abdominal frequency. In this study, GLM analysis considering data of both species showed that abdominal pumping frequency was dependent on (in order of importance) T_abdomen_, T_thorax_ and T_ambient_ (AIC = 137.18, *P*<0.0001; Table 1). Thus, the increase in abdominal temperature factored most prominently (Wald statistic = 17.91; *P*<0.001) in the increase of pumping abdominal frequency. GLM analysis for *S. sacer* showed that the abdominal pumping frequency was not related to any of the studied variables (Table 1). In the case of *S. cicatricosus*, T_ambient_ showed a tendency to be related with the abdominal pumping frequency (Table 1). These results also showed the existence of two different flight behaviours for both species although a greater amount of data at different T_ambient_ is needed to statistically confirm these results. For *S. cicatricosus*, the acquisition of an effective abdominal heat exchange allows flight during the hottest periods of the day when temperatures would otherwise be lethal for dung beetles with high endothermy [6],[7]. There is a lack of data in the literature regarding abdominal pumping behaviour during continuous flight because larger sized beetles are reluctant to participate in continuous flight. Although this mechanism of heat transfer from thorax to abdomen is common in large moths [7],[15], it had not been previously observed in beetles [14],[16]. The optimisation of experimental conditions and the use of thermography were crucial for obtaining measures of flight performance for prolonged periods of time (for both *Scarabaeus* species), which contributed to a clearer picture of the thermoregulatory mechanism during flight. In other dung beetles, such as *Geotrupes stercorarius* Linnaeus, abdominal pumping was assumed to be a mechanism for filling the tracheal system with new air and raising the concentration of oxygen needed for the flight muscles measured prior to flight [17]. In other insects, abdominal movement contributes to regulation of hemolymph flow and the effective ventilation of the tracheal system by a whole-body ensemble [18]. For example, in locusts and other flying insects, abdominal pumping facilitates the circulation of the hemolymph during flight [19],[20],[21],[22]. According to our results, abdominal pumping could also be considered as an adaptive mechanism that modulates heat flow between the thorax and the abdomen during continuous flight. Given that heat loss via convective cooling was avoided under laboratory conditions by controlling the environmental temperature, the main heat loss mechanisms in both *Scarabaeus* species could be considered internal mechanisms. These mechanisms facilitate heat transfer via hemolymph circulation in both species and possibly via respiratory evaporation heat loss in *S. cicatricosus*. As occurs in other insects [18],[19],[20],[21],[22] a combination between both internal mechanisms (hemolymph circulation and respiratory evaporation) could be the more efficient feature to explain heat loss in thermophilous insects, such as *S. cicatricosus*. Future studies using respirometry in different *Scarabaeus* species are needed to explore these possible internal mechanisms of heat loss in dung beetles.

**Table 1:** Results of the regression analysis using GLM between abdominal pumping and three explanatory variables: T_abdomen_, T_thorax_ and T_ambient_.

		Estimate	*SE*	*W*	*P*
Both species	Intercept	−4.95	0.63	61.09	<0.0001
	*T_abdomen_*	0.17	0.04	17.91	<0.0001
	*T_ambient_*	−0.04	0.02	4.45	0.0349
	*T_thorax_*	0.06	0.02	4.13	0.0420
	Scale	0.57	0.04	152.00	<0.0001
*Scarabaeus sacer*	Intercept	−1.56	0.93	2.81	0.0938
	*T_abdomen_*	0.05	0.06	1.01	0.4021
	*T_ambient_*	−0.006	0.01	0.25	0.6200
	*T_thorax_*	0.03	0.03	0.72	0.40
	Scale	0.22	0.02	76.00	<0.0001
*Scarabaeus cicatricosus*	Intercept	0.79	0.74	1.13	0.2870
	*T_abdomen_*	0.03	0.03	0.86	0.3538
	*T_ambient_*	−0.03	0.02	3.18	0.0746
	*T_thorax_*	0.01	0.02	0.37	0.5436
	Scale	0.39	0.04	76.00	<0.0001

SE: Standard Error.

W: Wald Statistic.

**Figure 5:**
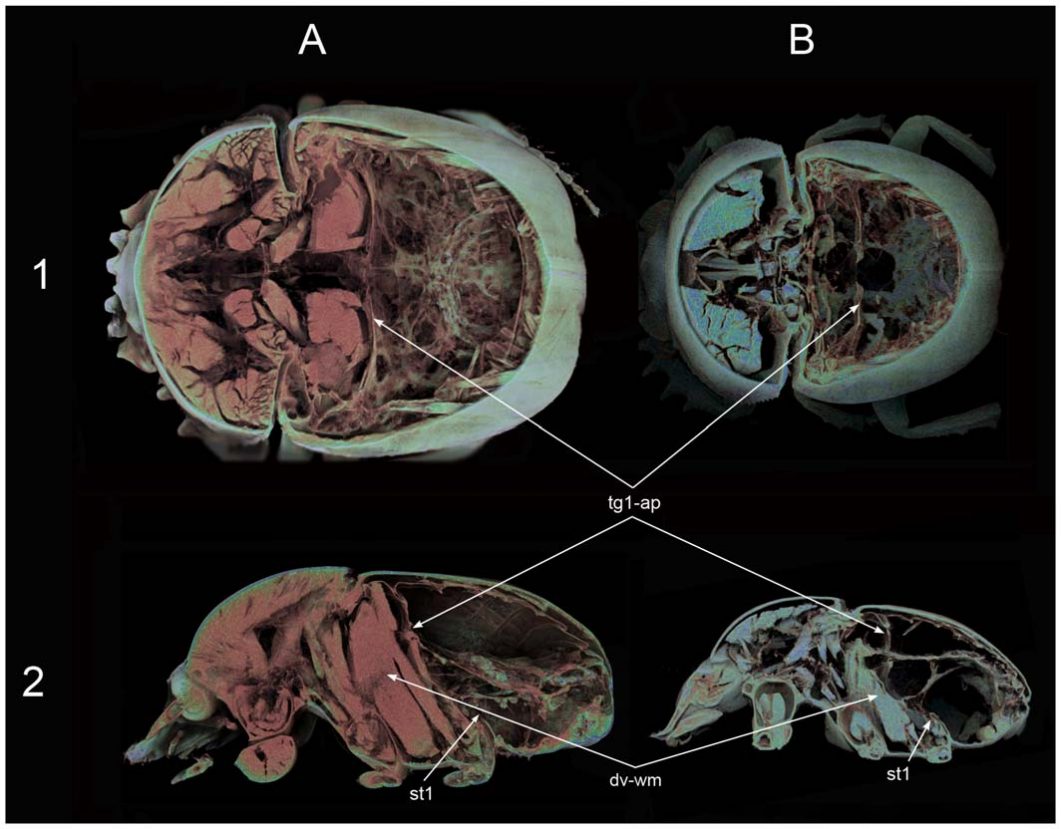
Micro-CT volume rendering reconstruction images of the internal anatomy. (A) *Scarabaeus sacer* and (B) *S. cicatricosus*. Dorsal (1) and lateral (2) views showing the cuticular membrane formed dorsally by the apodeme of the first abdominal terguite, being clearly evident the bigger development of the cuticular membrane of *S. sacer* in comparison with that of *S. cicatricosus*. Abbreviations: dv-wm: dorsoventral wing muscle; st1: 1^st^ abdominal sternite; tg1-ap: apodeme of the first abdominal terguite.

**Figure 6:**
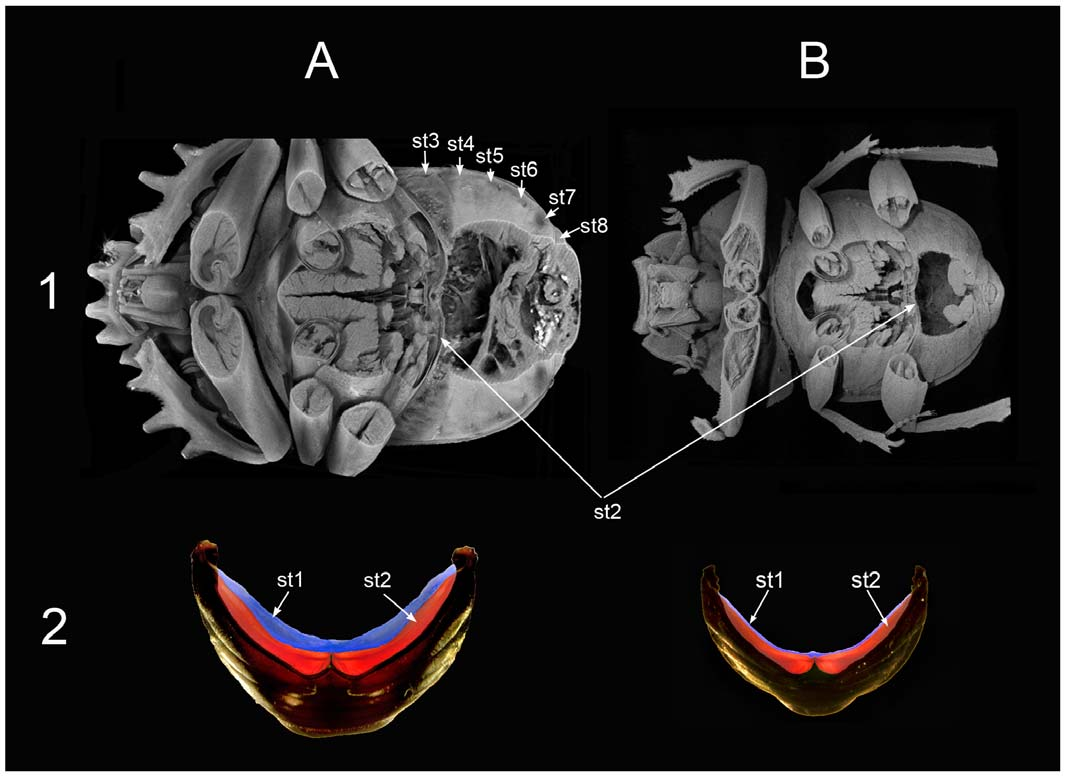
Micro-CT volume rendering reconstruction images (1) and from dissection (2) of the internal view. (A) *Scarabaeus sacer* and (B) *S. cicatricosus*. *Scarabaeus sacer* showed a greater development of the 1^st^ and 2^nd^ internal abdominal sternites than *S. cicatricosus*. Both internal sternites, but mainly the 1^st^, play a role as cuticular membrane in ventral position. Abbreviations: st1 to st8, correspond to all abdominal sternites consecutively numbered.

**Figure 7:**
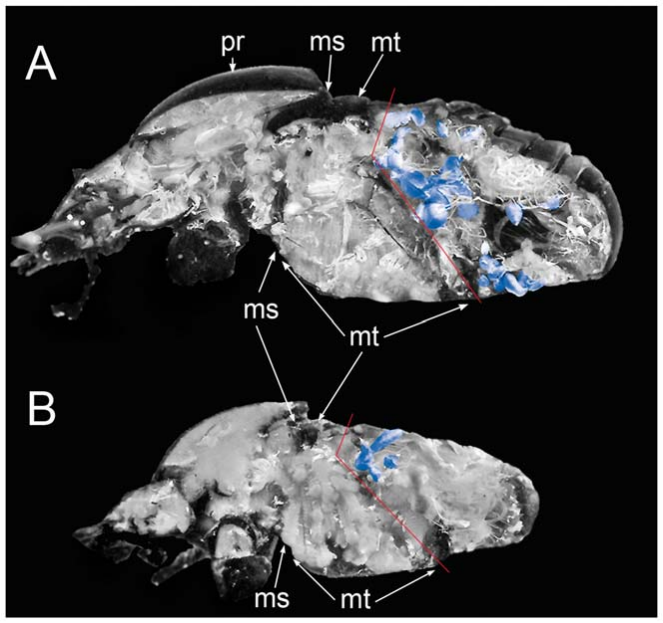
Distribution and development of abdominal air sacs. (A) *Scarabaeus sacer* and (B) *S. cicatricosus*. Abdominal air sacs were coloured in blue. Red lines indicate the separation between the metathorax and the abdomen. Abbreviations: pr: prothorax; ms: mesothorax; mt: metathorax.

Results obtained from X-ray computer microtomography (micro-CT), anatomical dissections and analysis of volume of the abdominal air sacs reinforce the idea of the existence of these two thermoregulation patterns. In line with Heinrich's observations in winter moths, *S. sacer* presents an anatomical design that is able to sequester heat within the meso- and metathorax to a greater extent than *S. cicatricosus*. This heat insulator implies the existence of two different types of structures; the first was revealed by micro-CT and consisted in a great development of both 1^st^ abdominal sternite and the 1^st^ abdominal terguite in *S. sacer* (Figures 5, 6 and Supporting Information S2 and S3). Both structures could generate a cuticle barrier between the metathorax and the abdomen capable of retaining the heat generated during pre-flight shivering and flight. Secondly, anatomical dissections revealed that *S. sacer* have a significant greater development of abdominal air sacs than *S. cicatricosus* (Fig. 7). These observations were corroborated by internal air volume measurements obtained for both species. *Scarabaeus sacer* showed a greater percentage of volume in its tracheal system than *S. cicatricosus* that suggesting the existence of a heat insulator mechanism that could be functionally more effective during flight performance (Table 2). Winter-flying endothermic moths (Cuculiinae) show a similar mechanism. This is crucial to warm up and fly at low T_ambient_ than other moths, because they can sequester heat within the thorax avoiding the dissipation by the abdomen [8].

**Table 2:** Tracheal and air sac volume (V_air_) of *Scarabaeus sacer* and *S. cicatricosus*.

	*Scarabaeus sacer*	*Scarabaeus cicatricosus*	*P*
V_air_ (ml)	1.44±0.37	0.14±0.05	<0.01
V_T_ (ml)	11.53±3.43	5.56±0.76	<0.01
%V_air_ *vs.* V_T_	11.66±2.75	2.79±1.46	<0.01
*N*	11	17	

Total body volume (V_T_) was measured using the function proposed by Radtke and Williamson (2005) based on total body mass.

These thermoregulatory mechanisms could have evolutionary implications to explain the relation between body size and endothermy, estimate the interspecific competition and/or thermal niche segregation between sympatric species, and even understand the causes of the geographical distribution of species and their response to climatic changes. The existence of a diversity of physiological responses as those showed in this study is key to exploring the consequences of directional selection in dung beetles. Competition for the dung of mammalian herbivores could have been an important factor in the evolution of endothermy and thermoregulation in roller dung beetles [23]. We suggest that the physiological thermal adaptations observed in these sympatric species could explain temporal and spatial segregations between co-occurring species. More information about the thermal niches of dung beetle species may provide us invaluable information to explain the ecology and the spatial distribution of dung beetles [6],[13],[24]. From the perspective of functional ecology and in the context of the theory of metabolic scaling [25],[26], our results suggest that these interspecific interactions are regulated by very different physiological mechanisms, which confer different thermal niches, limiting competition between species. This decrease in competition improves species richness and the composition of dung beetle assemblages. Moreover, a new perspective about the relation between body mass-body density (as extent of air sac volume *vs.* body volume), wing loading and thermoregulation suggest an interesting focus of research on physical boundaries in flying insects. In future studies, one might follow the occurrence of these thermoregulatory mechanisms across the phylogeny to examine the degree of variation of these mechanisms between phylogenetically close species and estimate the phylogenetic relevance of each mechanism using ancestral character-state reconstruction analysis. Little is known about the ecophysiological features of this group of animals, and we believe that further multidisciplinary research in this area could be an interesting nexus for the study of physiological diversity, functional ecology, evolutionary biology and biogeography.

## Materials and Methods

### Beetle collection

*Scarabaeus sacer* and *S. cicatricosus* are active from April to July in the Doñana National Park, UTM 29SQB21, Huelva (SE-Spain) [11]. Individuals of both species were collected in June 2010 and 2011 directly from the surface of horse droppings. To reduce the stress in the insects, all the individuals were maintained in plastic containers (60×40×40 cm) at 15°C until their arrival at the laboratory, where they were maintained at 20°C in a climate chamber until the experiments were conducted. Individuals were fed with cow dung free of pharmaceutical veterinary compounds. This work conforms to the Spanish legal requirements including those relating to conservation and welfare. Also, beetle collection was made with relevant permissions related to collection and field study in the Doñana National Park.

### Thermoregulatory behaviour during flight

Flight behaviour was analysed from the thermographic video sequences recorded with a FLIR ThermaCam P620 thermal infrared camera with a resolution of 640×480 pixels and a microbolometer Focal Plane Array detector with a spectral range of 7.5–13 µm and a thermal sensitivity of 0.06°C at 30°C. To ensure accuracy, the thermocamera was calibrated with the Standard Calibration service provided by FLIR Systems Inc. The ambient temperature (T_ambient_) was measured near each individual insect (less than one centimetre).

For free flight behaviour, beetles were fixed and suspended in the air using a retort stand with boss head and clamp. A plastic Pasteur pipette was fixed to clamp and pipette tips of 200 µl were used to join the support with the beetles using paraffin wax on the surface of the pronotum. Each beetle was released at a height of about 1.5 m from the floor. Prior to fixing, we selected individuals with a pre-flight behaviour characterised by the typical warm-up, which implies wing muscle vibrations (wing-shivering), muscle flexing exercises (forelegs and head, mainly) and occasional abdominal pumping movements [13],[16]. Pre-flight warm-up ensures that T_thorax_ is sufficiently high for takeoff and for maintaining continuous flight, independent of the T_ambient_. To ensure prolonged flight performance, we used low-level overhead lighting provided by daylight fluorescent tubes (60 W) and limiting any disturbance, vibration and odour that could alter the continuous flight. Measurements were made only when the beetle had been flying for at least 1 minute in the stable flight posture observed in the field [11] (Figure 1). In summary, we assume that all recorded body temperatures were dependent of the heat generation due to the activity of flight muscles and the formerly mentioned species specific heat dissipation flow capacities, and that the role played by convective (e.g. using airflows) or environmental heat transfers (e.g. IR radiation) can be considered negligible.

We used the ThermaCAM™ Researcher v 2.9 software [27] to record and analyse the obtained thermographic video sequences obtained. For the measurement of cuticle surface emissivities we used the thermocamera according to FLIR Systems Inc. manual procedure [27] and Rinaldi's suggestions [28]. Previously, we also measured the reflected temperature in the object parameters of the thermocamera. For this, we adjusted in the object parameters to ε = 1; then we placed the thermocamera against the cuticle surface of each species and writing the temperature reflected by an object (e.g. a finger of our hand). This temperature should always be measured any time that we want accurate measures of the object temperatures. In general, the temperature reflected ranged from 26 to 27°C and was noted in the object parameters accordingly.

For measure cuticle emissivity, we used electrical tape and soil as reference materials because their well known emissivities (ε = 0.95 and ε = 0.92, respectively). First, we measured the reflected temperature of the object as described below and the cuticles of each species and reference points (electrical tape and soil) were warmed up until 80°C using a digital precision heating plate SELECTA™ (+5 to 200°C; accuracy of 0.5°C). When cuticles and reference objects were warmed (after 15 min) at 80°C we have measured the temperature emitted by the electrical plate, setting previously a ε = 0.95 in the thermocamera. When temperature was noted, we set an area in the cuticle of each species and we changed manually the emissivity value (ε) of the thermocamera until the same temperature than reference was registered. To obtain a precise value of ε, we used the soil as a second reference point because we can warm more uniformly the fragments of cuticles maximizing the contact with the soil, avoiding the existence of a small air layer between cuticles and the heating plate. Thus, infrared emissivity was adjusted to 0.79 for *S. sacer* and 0.81 for *S. cicatricosus* according to their specific cuticle emissivities. Other object parameters supplied to measure temperature accurately were relative humidity = 65% and the distance between the object and the thermocamera (0.4 m).

### Analysis of thermal sequences

The slopes of T_thorax_ and T_abdomen_ against time for each individual were calculated by using temperature profiles (ThermaCAM™ Researcher v 2.9 software) recorded at 5 s intervals during a period of 1.5–2.0 min of continuous flight.

The variation in temperature from the thorax to the abdomen (T_thorax_–T_abdomen_) was measured as the difference between the temperature measured in the metathoracic plate and the third abdominal sternite (Fig. 1A). These measurements were made from the ventral view of the individual organisms to avoid any interference of leg movement and to ensure that the maximum temperature values recorded were generated by endothermy.

Abdominal pumping frequency was measured using IR sequences obtained from the lateral view. This permits the analysis of each individual thermal image of a sequence (around 30 images by second) to establish the maximum and minimum volume of the abdomen (Fig. 1B). When both limits were established in each sequence, the number of pulses/s was counted, and T_thorax_, T_abdomen_ and T_ambient_ were annotated accordingly.

### X-ray computer microtomography (micro-CT) study

Fresh collected specimens killed and preserved in 70% ethanol were used for the micro-CT study. Before to carry out the scanning, they were dehydrated in absolute alcohol for 24 h, and submerged for 48 h in Hexamethyldisilazane, overnight air dried, and an additional dry time of 24 h in a stove at 40°C.

Beetles were scanned with a micro-CT SkyScan 1172, by conducting an oversized scan (Number of connected scan were 4 for *S. cicatricosus* and 5 for *S. sacer*) with the following parameters: for *S. cicatricosus* an Aluminum filter was used (but no filter was used for *S. sacer*), Image Pixel Size (µm) = 9.90, Source Voltage (kV) = 73 (for *S. cicatricosus*) and 54 (for *S. sacer*), and a Source Current (µA) = 100. Images were reconstructed with ©*NRecon* software, with a Smoothing kernel = 2 (Gaussian). Later on, reconstructed images were “cleaned” with ©*CT-Analyser* by running a *Custom Processing Task List* (*Thresholding, Despeckle, ROI-Shrink-wrap, Reload, Bitwise operations* and *Save bitmaps*), obtaining a new series of reconstructed images. Images of the resulting series were corrected in their position with ©*Data viewer,* and saved as a definitive series, and finally the volume reconstruction images were obtained with the volume rendering software ©*CTvox* (for further information on micro-CT and software go to http://www.skyscan.be).

### Beetle dissections

Prior to the dissections, 16 beetles (N = 8, for each species) were killed with ethyl acetate. The longitudinal section of body was dissected from ice-cold beetles into ice-cold insect Ringer's solution. Air sacs were easily visible by flotation and a qualitatively approach was made.

### Air sac volume

Fresh collected specimens were killed with ethyl acetate. Air sac volume was measured using the water displacement method [29]. Beetle mass (fresh weight) was measured with a precision error of 0.1 mg. To measure the volume of the abdominal air sac, wings were removed with scissors to avoid retention of air below elytra and wings. Each beetle was then put in a 100 cm^3^ plastic syringe with a valve and filled with soapy distilled water. The air contained in the syringe was ejected and a vacuum pulled by hand was repeated six times to draw air out of the tracheae and the air sacs. The internal air of beetle was ejected after 3 pulses from the syringe. After six pulses no more air could be obtained into the beetle. Finally, the external water of the body was dried with filter paper and the beetle weighed, including the removing wings. The difference between initial and final masses was used as a measure of the volume of the respiratory system considering the density of water as 1 g/ml.

To obtain the percentage of volume of air sacs with respect to the total body volume we used the body weight measures converting them to volume according to Radtke and Williamson's function: V = (Total body weight/0.2)−0.02) [30].

### Statistical analyses

Temperature variation slopes against time were obtained from regression parameters calculated by the least squares method using non-parametric linear regressions [31]. T_thorax_, T_abdomen_ and T_ambient_ slopes for each species (n = 9, for each species) were compared using a non-parametric Kruskal-Wallis test with Conover-Inman post hoc test for pairwise comparisons [31]. A complementary analysis to test the individual relations between T_abdomen_ and T_ambient_, was developed using grouped linear regression with covariance analysis [31].

Heat flow rate between the thorax and the abdomen and the abdominal pumping frequency were compared between species using non-parametric Mann-Whitney U-test [31]. Finally, generalized linear models were used with a logarithmic link between abdominal pumping frequency (dependent variable) and the explanatory variables (T_thorax_, T_abdomen_ and T_ambient_). The best model was selected based on the Akaike information criterion (AIC; [32]).

Volume of air sacs and its percentage respect to total body volume was compared using Mann-Whitney U-test [31].

## Supporting Information

Supporting Information S1 Individual comparisons between slopes of T_abdomen_ and T_ambient_ during flight (median and confidence intervals), statistic values (t) and probabilities (P) for *Scarabaeus cicatricosus* (N = 27) and *S. sacer* (N = 24).
(DOC)

Supporting Information S2 Micro-CT reconstruction video of *Scarabaeus sacer*.
(MOV)

Supporting Information S3 Micro-CT reconstruction video of *Scarabaeus cicatricosus*.
(MOV)

## References

[1] May ML (1979). Insect thermoregulation.. Ann Rev Entomol.

[2] May ML, Kerkut G.A., Gilbert L.I. (1985). Thermoregulation.. Comprehensive Insect Physiology Biochemistry and Pharmacology.

[3] Prosser CL (1986). Adaptational Biology: Molecules to organisms.

[4] Cossins AR, Bowler K (1987). Temperature Biology of animals.

[5] Caveney S, Scholtz CH, McIntyre P (1995). Patterns of daily flight activity in onitine dung Beatles (Scarabaeinae: Onitini).. Oecologia.

[6] Verdú JR, Arellano L, Numa C, Micó E (2007). Roles of endothermy in niche differentiation for ball-rolling dung beetles (Coleoptera: Scarabaeidae) along an altitudinal gradient.. Ecol Entomol.

[7] Heinrich B (1993). Hot-blooded Insects: Strategies and Mechanisms of Thermoregulation.

[8] Heinrich B (1987). Thermoregulation by winter-flying endothermic moths.. J exp Biol.

[9] Hanski I, Cambefort Y, Hanski I, Cambefort Y (1991). Competition in dung beetles.. Dung Beetle Ecology.

[10] Bartholomew GA, Heinrich B (1978). Endothermy in African dung beetles during flight. Ball making and ball rolling.. J Exp Biol.

[11] Verdú JR, Díaz A, Galante E (2004). Thermoregulatory strategies in two closely related sympatric *Scarabaeus* species (Coleoptera: Scarabaeinae).. Physiol Entomol.

[12] Verdú JR, Arellano L, Numa C (2006). Thermoregulation in endothermic dung beetles (Coleoptera: Scarabaeidae): effect of body size and ecophysiological constraints in flight.. J Insect Physiol.

[13] Verdú JR, Lobo JM, Fattorini S. (2008). Ecophysiology of thermoregulation in endothermic dung beetles: ecological and geographical implications.. Insect Ecology and Conservation.

[14] Chown SL, Scholtz CH (1993). Temperature regulation in the nocturnal melolonthine *Sparrmannia flava*.. J Thermal Biol.

[15] Heinrich B (1996). Cooling off.. The Thermal Warriors.

[16] Merrick M (2004). Temperature regulation in burying beetles (Nicrophorus spp.: Coleoptera: Silphidae): effects of body size, morphology and environmental temperature.. J exp Biol.

[17] Krogh A, Zeuthen E (1941). The mechanism of flight preparation in some insects.. J exp Biol.

[18] Tartes U, Vanatoa A, Kuusik A (2002). The insect abdomen—a heartbeat manager in insects?. Comp Biochem Physiol A.

[19] Weis-Fogh T (1967). Respiration and tracheal ventilation in locusts and other flying insects.. J exp Biol.

[20] Casey TM (1976). Flight energetics in sphinx moths: heat production and heat loss in *Hyles lineata* during free flight.. J exp Biol.

[21] Prange HD (1990). Temperature regulation by respiratory evaporation in grasshoppers.. J exp Biol.

[22] Lighton JRB (1994). Discontinuous ventilation in terrestrial insects.. Physiol Zool.

[23] Heinrich B, Bartholomew GA (1979). Roles of endothermy and size in inter- and intraspecific competition for elephant dung in an African dung beetle, *Scarabaeus laevistriatus*.. Physiol Zool.

[24] Gaston KJ, Chown SL (1999). Elevation and climatic tolerance: a test using dung beetles.. Oikos.

[25] West GB, Brown JH, Enquist BJ (1999). The fourth dimension of life: Fractal geometry and allometric scaling of organisms.. Science.

[26] Brown JH, Gillooly JF, Allen AP, Savage VM, West GB (2004). Toward a metabolic theory of ecology.. Ecology.

[27] Flir Systems Ltd (2007). ThermaCAM™ Researcher v 2.9 software..

[28] Rinaldi R (2002). Emissivity: the common problem for all thermographers.. Inframation.

[29] Bartholomew GA, Barnhart MC (1984). Tracheal gases, respiratory gas exchange, body temperature and flight in some tropical cicadas.. J Exp Biol.

[30] Radtke M, Williamson G (2005). Volume and linear measurements as predictors of dung beetle (Coleoptera: Scarabaeidae) biomass.. Ann Entomol Soc Am,.

[31] StatsDirect Ltd (2005).

[32] Akaike H (1973). A new look at the statistical model identification.. IEEE Trans Automatic Control.

