# Supplementary material for: Evidence of Different Thermoregulatory Mechanisms between Two Sympatric Scarabaeus Species Using Infrared Thermography and Micro-Computer Tomography
Source: PLoS One. 2012 Mar 19;7(3):e33914. doi: 10.1371/journal.pone.0033914 (PMC3307782; doi:10.1371/journal.pone.0033914)
Supplement: Supporting Information S1 — Individual comparisons between slopes of Tabdomen and Tambient during flight (median and confidence intervals), statistic values (t) and probabilities (P) for Scarabaeus cicatricosus (N = 27) and S. sacer (N = 24). (DOC) [file pone.0033914.s001.doc]

**S1**: Individual comparisons between slopes of T_abdomen_ and T_ambient_ during flight (median and confidence intervals), statistic values (t) and probabilities (P) for *Scarabaeus cicatricosus* (N = 27) and *S. sacer* (N = 24).

| Species | Difference between slopes (95% CI) | t | P |
| --- | --- | --- | --- |
| *Scarabaeus cicatricosus* | 0.752 (0.675 to 0.829) | 19.328 | <0.0001 |
|  | 0.089 (0.079 to 0.098) | 18.900 | <0.0001 |
|  | 0.733 (0.637 to 0.828) | 15.408 | <0.0001 |
|  | 0.084 (0.072 to 0.096) | 13.760 | <0.0001 |
|  | 0.529 (0.440 to 0.619) | 11.856 | <0.0001 |
|  | 0.391 (0.321 to 0.461) | 11.159 | <0.0001 |
|  | 0.688 (0.517 to 0.859) | 8.146 | <0.0001 |
|  | 0.710 (0.530 to 0.890) | 7.876 | <0.0001 |
|  | 0.625 (0.458 to 0.792) | 7.563 | <0.0001 |
|  | 0.383 (0.280 to 0.486) | 7.470 | <0.0001 |
|  | 0.378 (0.275 to 0.480) | 7.374 | <0.0001 |
|  | 0.564 (0.405 to 0.722) | 7.068 | <0.0001 |
|  | 0.079 (0.053 to 0.105) | 6.145 | <0.0001 |
|  | 0.741 (0.465 to 1.018) | 5.452 | <0.0001 |
|  | 0.794 (0.491 to 1.097) | 5.345 | <0.0001 |
|  | 0.516 (0.300 to 0.731) | 4.786 | <0.0001 |
|  | 0.341 (0.197 to 0.485) | 4.732 | <0.0001 |
|  | 0.489 (0.281 to 0.697) | 4.713 | <0.0001 |
|  | 0.335 (0.174 to 0.495) | 4.142 | <0.0001 |
|  | 0.404 (0.204 to 0.604) | 4.056 | <0.001 |
|  | 0.530 (0.263 to 0.797) | 4.036 | <0.001 |
|  | 0.375 (0.169 to 0.582) | 3.655 | <0.001 |
|  | 0.079 (0.026 to 0.131) | 3.210 | <0.001 |
|  | 0.215 (0.074 to 0.356) | 3.061 | <0.01 |
|  | 0.199 (0.057 to 0.341) | 2.806 | <0.01 |
|  | 0.046 (-0.134 to 0.225) | -0.506 | 0.614 |
|  | 0.135 (-0.143 to 0.412) | -0.980 | 0.333 |
|  |  |  |  |
| *Scarabaeus sacer* | 0.066 (-0.083 to 0.215) | -0.900 | 0.375 |
|  | 0.082 (-0.108 to 0.241) | -0.863 | 0.392 |
|  | 0.131 (-0.201 to 0.464) | -0.861 | 0.406 |
|  | 0.048 (-0.161 to 0.257) | -0.462 | 0.647 |
|  | 0.011 (-0.273 to 0.294) | -0.077 | 0.939 |
|  | 0.014 (-0.396 to 0.424) | -0.074 | 0.942 |
|  | 0.013 (-0.270 to 0.296) | 0.094 | 0.926 |
|  | 0.018 (-0.195 to 0.231) | 0.167 | 0.868 |
|  | 0.036 (-0.330 to 0.403) | 0.217 | 0.832 |
|  | 0.034 (-0.174 to 0.241) | 0.330 | 0.744 |
|  | 0.144 (-0.422 to 0.711) | 0.542 | 0.596 |
|  | 0.169 (-0.321 to 0.659) | 0.724 | 0.478 |
|  | 0.140 (-0.245 to 0.525) | 0.758 | 0.457 |
|  | 0.114 (-0.176 to 0.405) | 0.805 | 0.427 |
|  | 0.075 (-0.093 to 0.243) | 0.905 | 0.372 |
|  | 0.055 (-0.062 to 0.172) | 0.940 | 0.351 |
|  | 0.190 (-0.122 to 0.502) | 1.228 | 0.226 |
|  | 0.127 (-0.071 to 0.325) | 1.306 | 0.201 |
|  | 0.178 (-0.061 to 0.416) | 1.509 | 0.139 |
|  | 0.091 (-0.019 to 0.201) | 1.680 | 0.101 |
|  | 0.139 (-0.012 to 0.296) | 1.780 | 0.083 |
|  | 0.179 (0.008 to 0.367) | 1.905 | 0.061 |
|  | 0.166 (0.007 to 0.326) | 2.106 | 0.041 |
|  | 0.499 (0.317 to 0.682) | 5.453 | <0.0001 |
